# Differential Effects of Small Molecule Inhibitors on the Intracellular Chlamydia Infection

**DOI:** 10.1128/mbio.01076-22

**Published:** 2022-06-15

**Authors:** Karissa J. Muñoz, Ming Tan, Christine Sütterlin

**Affiliations:** a Department of Developmental and Cell Biology, University of California, Irvine, California, USA; b Department of Microbiology and Molecular Genetics, University of California, Irvine, California, USA; c Department of Medicine, University of California, Irvine, California, USA; University of Maryland School of Medicine

**Keywords:** developmental cycle, inclusion, replication, RB-to-EB conversion, progeny assay

## Abstract

Chlamydia are obligate intracellular bacteria that reside within a membrane-bound compartment called the chlamydial inclusion inside a eukaryotic host cell. These pathogens have a complex biphasic developmental cycle, which involves conversion between a replicating, but noninfectious, reticulate body (RB) and an infectious elementary body (EB). Small molecule inhibitors have been reported to have deleterious effects on the intracellular Chlamydia infection, but these studies have typically been limited in terms of assays and time points of analysis. We compared published and novel inhibitors and showed that they can differentially alter inclusion size, chlamydial number and infectious EB production, and that these effects can vary over the course of the intracellular infection. Our results provide the justification for analysis with multiple assays performed either at the end of the infection or over a time course. We also show that this approach has the potential to identify the particular step in the developmental cycle that is impacted by the inhibitor. We furthermore propose that the magnitude of inhibitor-induced progeny defects are best quantified and compared by using a new value called maximal progeny production (Progeny_max_). As a demonstration of the validity of this systematic approach, we applied it to inhibitors of Akt and AMPK, which are host kinases involved in lipid synthesis and cholesterol trafficking pathways. Both inhibitors reduced EB production, but Akt disruption primarily decreased RB-to-EB conversion while AMPK inhibition paradoxically enhanced RB replication.

## INTRODUCTION

Chlamydia is the most common bacterial cause of sexually transmitted infection in the United States. Over 1.8 million cases of chlamydial infection are reported to the CDC each year, and this number is continually increasing (1). There is no vaccine to prevent infection, which leaves surveillance testing and antibiotic treatment as the main management strategies to combat this public health challenge (2). Small molecules have been reported to have anti-Chlamydia activity *in vitro*. While these inhibitors have the potential to be developed into antibiotics, they are also powerful tools for mechanistic studies of this wide-spread infection.

Chlamydia are obligate intracellular bacteria that replicate within an infected host cell via an unusual biphasic developmental cycle (3). The infection begins with uptake of an elementary body (EB), the infectious form of the bacterium, into a eukaryotic host cell. Around 2- to 8-hours post infection (hpi), the EB converts into a reticulate body (RB), which is the metabolically active, dividing, but noninfectious form of Chlamydia. After multiple rounds of RB replication, RBs asynchronously convert into EBs, which are released from the host cell either by lysis or inclusion extrusion to infect new host cells (4). The length of the developmental cycle varies with Chlamydia spp., but for Chlamydia trachomatis, EBs are first produced at about 24 hpi and the developmental cycle lasts about 48 hours (h).

In an infected host cell, RBs and EBs are contained within a membrane-bound vacuole called the chlamydial inclusion. This compartment grows over time until it occupies most of the host cytoplasm and can be readily detected by light microscopy. Its membrane is composed of lipids and cholesterol, which are acquired from the host cell (5–8), but the mechanisms by which Chlamydia hijacks these molecules are not completely understood. The inclusion membrane also contains approximately 50 chlamydial inclusion membrane proteins (Incs) that mediate interactions with host organelles (9–12).

During the intracellular Chlamydia infection, there is a 1,000-fold increase in both the number of chlamydiae and the volume of the inclusion (13). Detailed quantitative and volumetric analyses have shown a tight correlation between chlamydial number and inclusion size over the entire course of a wild-type infection (13). Similarly, a correlation between chlamydial genome copy number and inclusion growth has been reported (14). As inclusion size can be readily monitored by immunofluorescence microscopy, it has often been used as a surrogate measure for the overall infection.

A number of small molecules have been reported to have anti-chlamydial activity in a cell culture model of Chlamydia infection, as determined by measurements of inclusion size by immunofluorescence microscopy and/or EB production in progeny assays. Another, less frequently used assay is the quantification of chlamydial genome copy number by qPCR, which determines the total number of bacteria, but does not distinguish between RBs and EBs. A few studies have conducted this analyses over a time course (15–20), but by and large, the effects of inhibitors have been examined with one or two assays at one or two time points in the intracellular infection. The dynamic nature of this biphasic developmental cycle raises several questions: (i) What are the best assays and time points to measure the effects of an inhibitor on the intracellular infection? (ii) Do analyses at one or two time points accurately reflect the overall effects on the infection? (iii) How can an inhibitor be analyzed if it alters the length of the developmental cycle? (iv) How can the effects of different inhibitors be compared?

To answer these questions, we systematically studied how several published and novel chlamydial inhibitors alter the chlamydial infection. For each inhibitor, we determined the time point corresponding to the end of the infection, which allowed us to measure changes in the length of the developmental cycle. We then quantified inhibitor-induced alterations of inclusion growth, chlamydial replication, and progeny production over the course of the intracellular infection. Our experiments showed that (i) small molecule inhibitors can have temporal and differential effects on the intracellular infection, (ii) the magnitude of a progeny defect can be quantified and compared through a new value called Progeny_max_, and (iii) our approach can provide mechanistic insights into the specific step in the Chlamydia developmental cycle that is disrupted by an inhibitor.

## RESULTS

**FIG 1:**
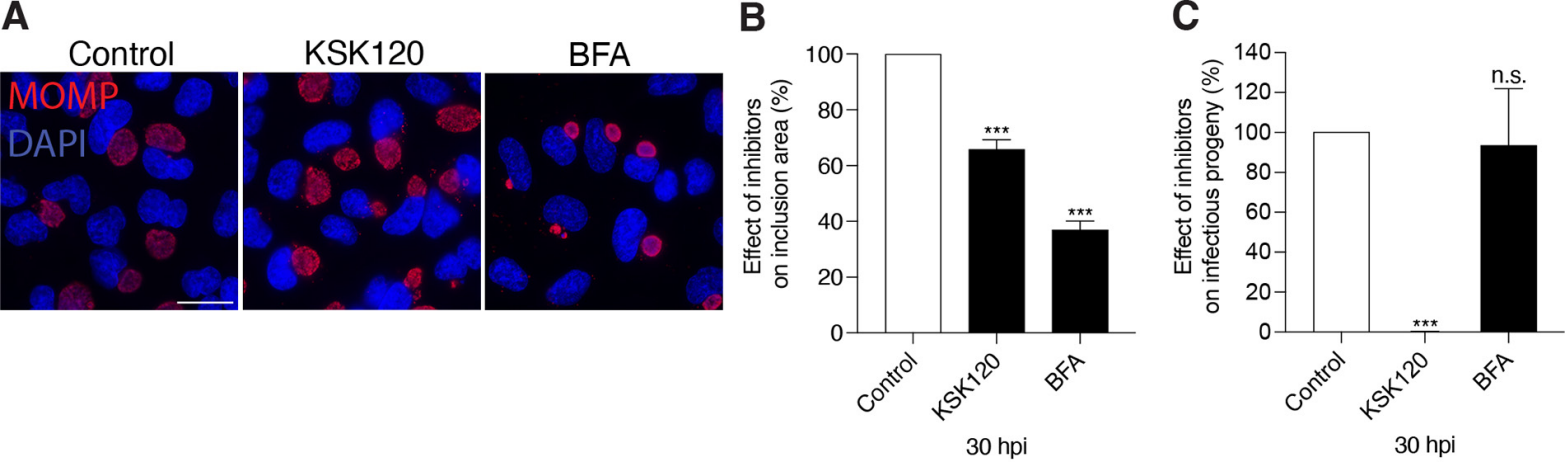
Inhibitors have discordant effects on the Chlamydia infection. (A) Immunofluorescence images of Chlamydia trachomatis L2-infected HeLa cells treated with KSK120 or Brefeldin A (BFA) inhibitors starting at 1 hpi. Samples were fixed 30 hpi. To visualize the chlamydiae, samples were stained with antibodies to MOMP (red), while chlamydial and host DNA was detected with DAPI (blue). Scale bar is 10 μm. (B) For each sample, the average inclusion area from 33 to 34 inclusions was determined. The data are expressed as percentage of control. The results from three independent experiments are shown. The data are presented as means ± SE (*n *= 3); ***, *P < *0.001. (C) A progeny assay was performed to determine the number of infectious EBs for control and inhibitor-treated HeLa cells at 30 hpi. Values of the inhibitor-treated conditions were normalized to their respective controls and are expressed as a percentage. Data are presented as means ± SE (*n *= 3); ***, *P < *0.001; n.s., data not statistically significant.

We began our study with a head-to-head comparison of two small molecule inhibitors that alter the intracellular Chlamydia infection. KSK120 is an inhibitor of bacterial glucose metabolism that has been reported to delay inclusion growth and decrease progeny production (21, 22). Brefeldin A (BFA), a known inhibitor of host cell protein transport, has been found to produce smaller inclusions without disrupting progeny production (7, 8). We infected HeLa cells with C. trachomatis serovar L2 and treated with the respective inhibitor starting at 1 hpi at concentrations that were nontoxic to the host cell and did not affect infection efficiency (Fig. S1). At 30 hpi, which is a late time in the intracellular infection, we measured inclusion size by immunofluorescence microscopy and quantified the number of infectious EBs through progeny assays. Both KSK120 and BFA produced smaller inclusions compared with untreated controls (Fig. 1A and B). However, KSK120 caused a large, 277-fold reduction in progeny, while BFA had no significant effect (Fig. 1C); both findings are consistent with published data (8, 21). However, these discordant effects on inclusion size and progeny demonstrate how a single assay may not adequately measure the effects of an inhibitor on the Chlamydia infection.

10.1128/mbio.01076-22.1 FIG S1 Inhibitor treatment does not alter infection efficiency in HeLa cells. HeLa cells were infected on coverslips and treated with inhibitors from 1 to 48 hpi. Infection efficiency was determined by calculating the percentage of HeLa cells with an inclusion. Data are presented as means ± SE (*n *= 3); n.s., data not statistically significant. Download FIG S1, TIF file, 0.8 MB. Copyright © 2022 Muñoz et al. 2022 Muñoz et al. https://creativecommons.org/licenses/by/4.0/ This content is distributed under the terms of the Creative Commons Attribution 4.0 International license.

**FIG 2:**
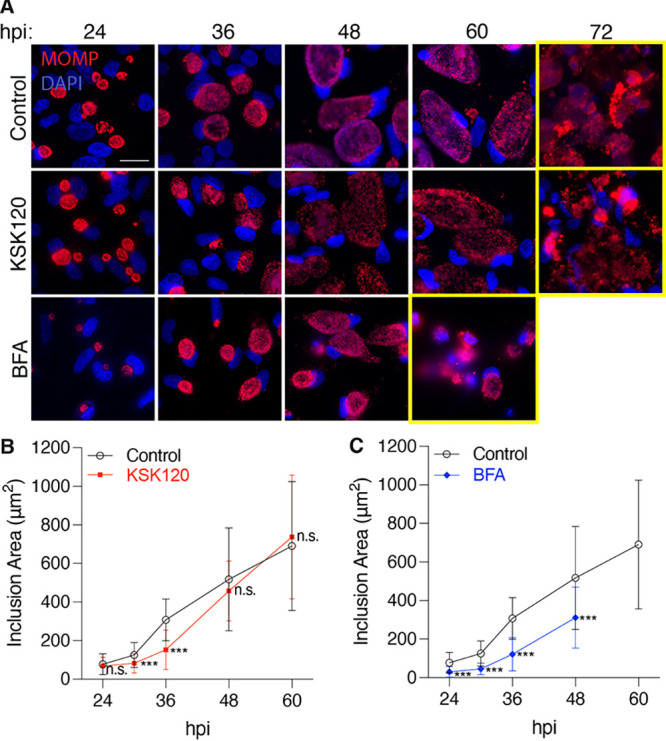
The effects of inhibitors on inclusion size can change during the time course of infection. (A) Immunofluorescence images of Chlamydia trachomatis L2-infected HeLa cells treated with KSK120 or BFA inhibitors starting at 1 hpi. Samples were fixed at the times indicated and stained with antibodies to MOMP (red) and DAPI (blue). Scale bar is 10 μm. Yellow boxes indicate a time point when wide-spread inclusion and host cell lysis is observed; the time point when lysis is observed in few cells is what we refer to as the “lysis onset.” (B) The average area of 100 inclusions was measured for KSK120-treated cells at each time point. The data from one representative experiment are presented as means ± SD; ***, *P < *0.001; n.s., data not statistically significant. (C) Same as in (B) but for BFA-treated cells. The data from one representative experiment are shown as means ± SD; ***, *P < *0.001.

We next examined the consequences of KSK120 and BFA treatments on inclusion size over the course of the developmental cycle (Fig. 2). Inclusions in KSK120-treated cells were initially smaller, but grew to a similar size as control cell inclusions (Fig. 2A and B), consistent with previous reports (21, 22). In contrast, BFA treatment caused a 2-fold reduction in inclusion size at all time points (Fig. 2A and B). These results demonstrate that small molecule inhibitors can have effects on inclusion growth that may not be uniform over the course of the intracellular infection. Moreover, they revealed the limitations of a single time point analysis and the hazards of extrapolating from measurements taken partway through the developmental cycle.

Our microscopy-based time course analysis also revealed that inhibitors can alter the length of the chlamydial developmental cycle. For control or KSK120 treatment, intact inclusions were visible up to 60 hpi, followed by widespread lysis of the inclusion and the host cell (Fig. 2A). In contrast, inclusions in BFA-treated cells only remained intact up to 48 hpi (Fig. 2A) and then underwent lysis. Thus, BFA appears to shorten the developmental cycle. These data raise the question of when to analyze and compare the effects of inhibitors if the length of the developmental cycle is altered.

**FIG 3:**
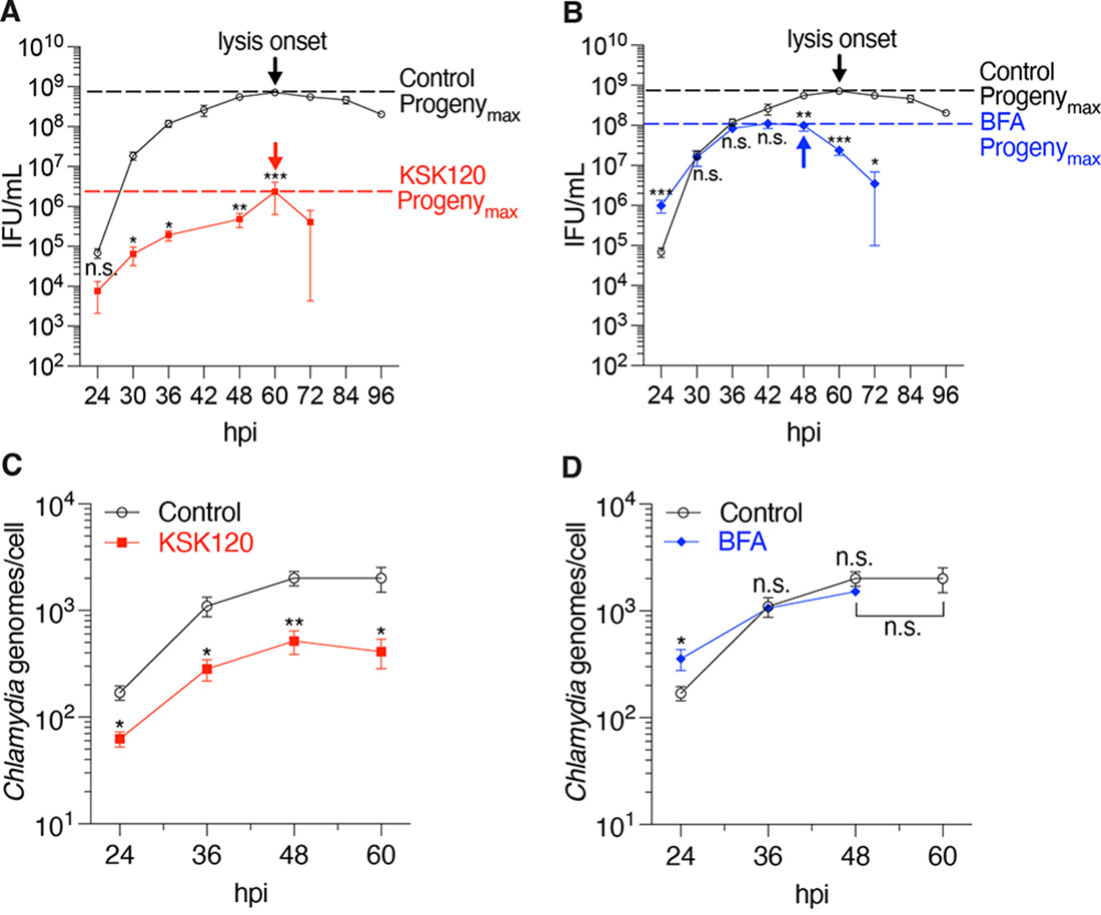
Progeny defects can result from effects on different steps of the Chlamydia developmental cycle. (A) A progeny assay time course was used to determine the maximal number of infectious progeny (Progeny_max_) yielded from intact inclusions in KSK120-treated cells. The onset of lysis (lysis onset) is indicated by arrows. The horizontal dashed lines represent Progeny_max_, which coincides with the onset of lysis. Data are presented as means ± SE (*n *= 3); *, *P < *0.05; **, *P ≤ *0.01; ***, *P < *0.001; n.s., data not statistically significant. (B) Same as in (A), but for BFA-treated cells. Data are presented as means ± SE (*n *= 3); *, *P < *0.05; **, *P ≤ *0.01; ***, *P < *0.001; n.s., data not statistically significant. (C) The number of chlamydial genomes for infected KSK120-treated HeLa cells was determined by qPCR at the indicated time points. Data are presented as means ± SE (*n *= 3). *, *P < *0.05; **, *P ≤ *0.01. (D) Same as in (C), but for BFA-treated HeLa cells. Data are presented as means ± SE (*n *= 3). *, *P < *0.05; n.s., data not statistically significant.

To address this issue, we measured infectious progeny at 6- to 12-h intervals over the time course of the infection to generate a one-step growth curve. For all inhibitors examined, progeny production increased to a peak and then gradually decreased at very late times (Fig. 3A and B). However, peak progeny production, which we called “Progeny_max_,” varied in time, occurring at 60 hpi for control and KSK120-treated cells, but at 48 hpi for BFA-treated cells. For all inhibitors, Progeny_max_ coincided with the time point when we first detected host cell lysis, but most of the infected cells were still intact by light microscopy (data not shown). Compared with untreated control cells, Progeny_max_ for KSK120- and BFA-treated cells were reduced by 83-fold (Fig. 3A) and 9-fold (Fig. 3B), respectively.

As a progeny defect could be due to effects on chlamydial replication or RB-to-EB conversion, we determined the number of chlamydial genomes by qPCR to assess possible effects on chlamydial replication. KSK120 treatment decreased the total number of chlamydiae by 16-fold, while BFA had no significant effect (Fig. 3C and D). Comparing the effects on chlamydial replication and infectious progeny production at the time point of Progeny_max_ revealed that KSK120 has deleterious effects on both RB replication and RB-to-EB conversion (Fig. 3A and C) (21, 22). BFA, in contrast, had no effect on RB replication but primarily reduced progeny production, consistent with a defect in RB-to-EB conversion (Fig. 3B and D). Overall, our analysis shows that KSK120 and BFA disrupt different steps in the developmental cycle.

**FIG 4:**
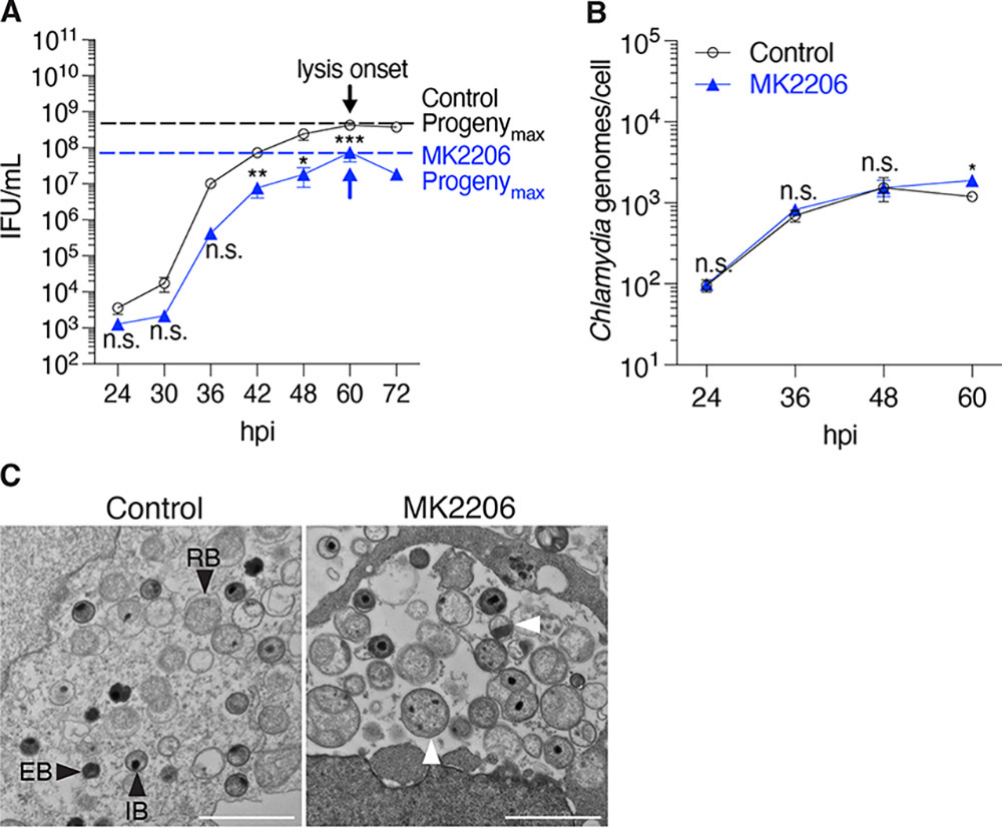
MK2206 may cause progeny defects by inhibiting RB-to-EB conversion. (A) Measurements of progeny over the time course of infection were used to determine Progeny_max_ of MK2206-treated cells. Arrows indicate the onset of inclusion and cell lysis (lysis onset), which is the time point when Progeny_max_ was determined. Data are presented as means ± SE (*n *= 3); *, *P < *0.05; **, *P ≤ *0.01; ***, *P < *0.001; n.s., data not statistically significant. (B) The number of chlamydial genomes for infected MK2206-treated HeLa cells was determined by qPCR at the indicated time points. Data are presented as means ± SE (*n *= 3). *, *P < *0.05; n.s., data not statistically significant. (C) Electron micrographs of infected cells treated with DMSO or MK2206 from 1 to 48 hpi. Scale bar, 2 μm. Chlamydial developmental forms are indicated: EB, elementary body; IB, intermediate body; RB, reticulate body. Abnormal IBs with multiple dense chromatin foci are indicated with white arrowheads.

We then applied our systematic approach to two novel inhibitors that block synthesis and transport of host lipids and cholesterol, which are incorporated into both the inclusion and the chlamydial membrane (5, 6, 12). As the Golgi is a known source of lipids for the Chlamydia infection (7, 8, 10, 12), we used MK2206 to inhibit Akt kinase activity, which is critical for lipid trafficking through the Golgi (23). MK2206 treatment did not alter inclusion growth (Fig. S2), chlamydial replication (Fig. 4B), or the length of the developmental cycle (Fig. 4A; Fig. S2). However, at the time point of Progeny_max_, MK2206 treatment caused a 10-fold reduction in progeny (Fig. 4A). Furthermore, EM analysis revealed similar numbers of RBs but significantly fewer EBs than control cells. We also noted unusual-looking IBs, which are RBs in the process of converting into EBs, that were abnormally large with multiple dense nuclei (Fig. 4C). Together these data suggest that Akt kinase activity is important for RB-to-EB conversion.

10.1128/mbio.01076-22.2 FIG S2 Inclusion growth for infected MK2206 or CompC-treated cells. (A) Immunofluorescence images of Chlamydia trachomatis L2-infected HeLa cells treated with DMSO, MK2206, or CompC starting at 1 hpi. Samples were fixed at the times indicated and stained with antibodies to MOMP (red) and DAPI (blue). Scale bar is 10 μm. (B) The average inclusion area from 100 inclusions was measured for each condition at each time point. The data from one representative experiment are presented as means ± SD;. *, *P < *0.05; ***, *P < *0.001; n.s., data not statistically significant. Download FIG S2, TIF file, 1.3 MB. Copyright © 2022 Muñoz et al. 2022 Muñoz et al. https://creativecommons.org/licenses/by/4.0/ This content is distributed under the terms of the Creative Commons Attribution 4.0 International license.

**FIG 5:**
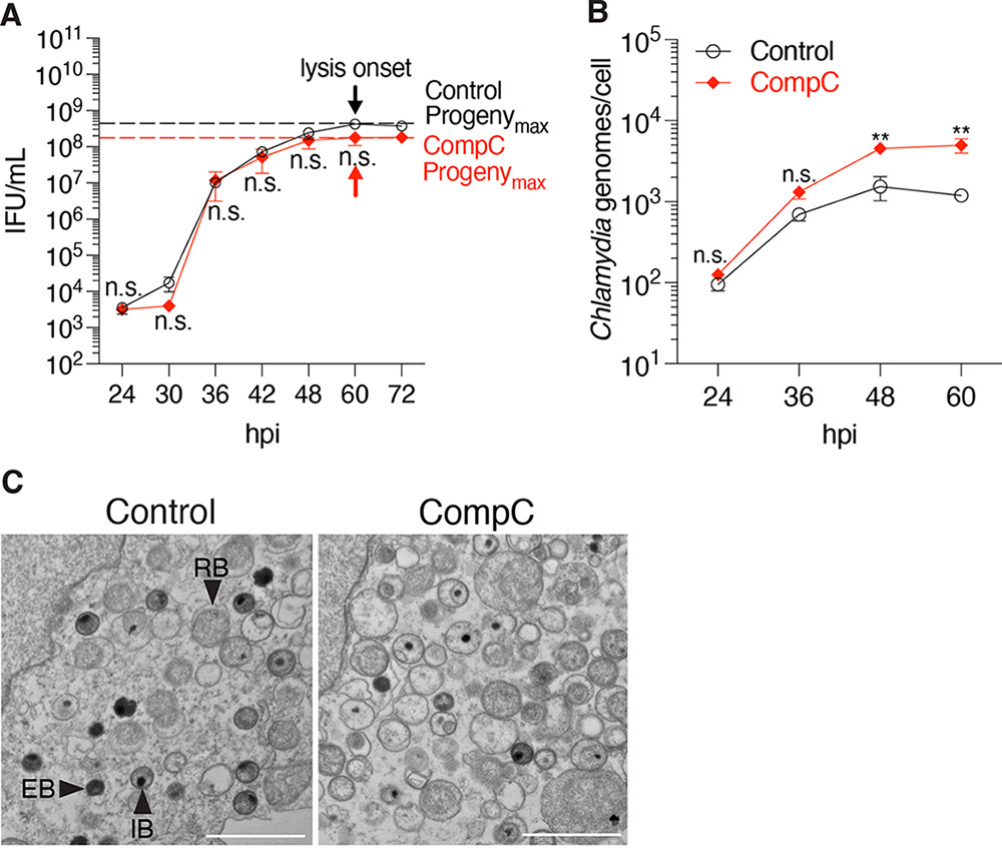
Compound C (CompC) treatment promotes chlamydial replication. (A) Measurements of progeny during the time course of infection was used to determine the Progeny_max_ of CompC-treated cells. Arrows indicate the onset of inclusion lysis, at which Progeny_max_ was measured. Data are presented as means ± SE (*n *= 3); n.s., data not statistically significant. (B) The number of chlamydial genomes for infected CompC-treated HeLa cells was determined by qPCR at the indicated time points. Data are presented as means ± SE (*n *= 3). **, *P ≤ *0.01; n.s., data not statistically significant. (C) Electron micrographs of infected cells treated with CompC from 1 to 42 hpi. Scale bar, 2 μm. Chlamydial developmental forms are indicated: EB, elementary body; IB, intermediate body; RB, reticulate body.

We also tested Compound C, an inhibitor of the host cell kinase AMPK, which limits host cell cholesterol synthesis and promotes the breakdown of this important membrane component (24). Compound C did not alter the length of the chlamydial developmental cycle and slightly increased inclusion size (Fig. 5A; Fig. S2). However, at the time point of Progeny_max_ (60 hpi), we measured a 4-fold increase in chlamydial genomes by qPCR (Fig. 5B), which is consistent with the increased number of chlamydiae that we detected by EM (Fig. 5C). Thus, Compound C appears to enhance chlamydial replication. However, as there was no corresponding increase in EB production, we conclude that Compound C may also have an inhibitory effect on RB-to-EB conversion.

**FIG 6:**
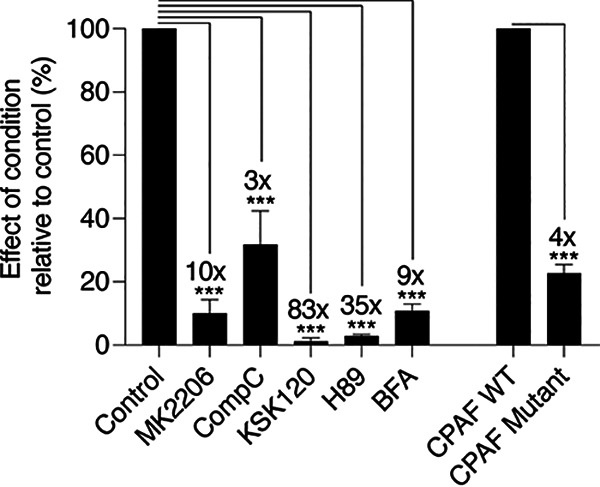
Experimental manipulations can alter Progeny_max_ to different degrees. For each inhibitor, Progeny_max_ was normalized to untreated control cells and expressed as a percentage of control. Similarly, Progeny_max_ of CPAF RST17 loss-of-function mutant was normalized to the parental CPAF WT strain. The fold change reduction in Progeny_max_ is indicated above the error bars. Data are presented as means ± SE (*n *= 3); ***, *P < *0.001.

To put these results in context, we used Progeny_max_ to compare the magnitude of the anti-chlamydial effects of these inhibitors (Fig. 6). For example, MK2206 and BFA each caused an about 10-fold decrease in progeny, while KSK120 and another published chlamydial inhibitor, H89, reduced progeny by 83- and 35-fold, respectively (17). These effects were much more severe than what we observed for Compound C, which produced a modest 3-fold reduction in progeny, in the same range as the CPAF mutant, RST17 (Fig. 6), a well-characterized C. trachomatis loss-of-function mutant (25). This analysis demonstrates how Progeny_max_ can be used to quantify and compare the effects of different inhibitors and other manipulations on a productive chlamydial infection.

**FIG 7:**
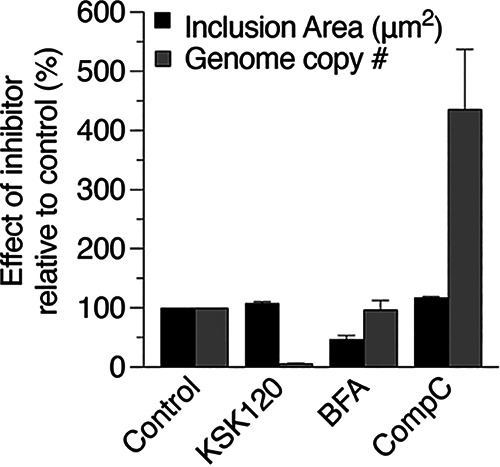
Inclusion size and chlamydiae numbers do not always correlate. Inclusion areas and numbers of chlamydial genomes for inhibitor-treated samples were determined at the time of lysis onset. For control, KSK120, and Compound C lysis onset was at 60 hpi. For BFA, this time point was 48 hpi. Values were normalized to untreated controls (control = 100%) and are expressed as a percentage to indicate the relative effect of the inhibitors. Data are presented as means ± SE (*n *= 3).

Finally, we used our data to examine the relationship between inclusion size and chlamydial number (Fig. 7). At the time point of lysis onset, KSK120-treated cells had inclusions of normal size but few chlamydiae. In contrast, inclusions in BFA-treated cells were smaller, but chlamydial number was unaffected. Compound C caused a completely different effect by increasing chlamydial number without greatly affecting inclusion size. These findings demonstrate that inhibitors can disrupt the linear relationship between inclusion growth and the increase in chlamydial number observed during a wild-type C. trachomatis infection (13) in unpredictable ways.

## DISCUSSION

In this study, we present a systematic approach to analyze and compare the effects of small molecule inhibitors on the intracellular Chlamydia infection. We show that inhibitors can produce discordant effects on inclusion growth, chlamydial replication, and infectious progeny production, providing strong justification for analyzing an inhibitor with multiple assays. We also report that inhibitors may not have uniform effects throughout the developmental cycle, raising the issue of when to analyze inhibitor treatment during the 48- to 72- hour long Chlamydia developmental cycle. As a solution, we introduced Progeny_max_ as a new measure that allows the effects of inhibitors on a productive Chlamydia infection to be quantified and compared. We also demonstrate how our approach can reveal the step in the developmental cycle that is affected by an inhibitor and thereby provide novel mechanistic insights into the regulation of this infection.

Our experiments reveal the limitations of analyzing the effects of an inhibitor at one or two time points partway through the intracellular infection. For example, KSK120 produced smaller inclusions early on but then grew to the same size as control inclusions, consistent with a delay, rather than a block, in inclusion growth (Fig. 2) (22). With BFA and MK2206, progeny production was normal at early time points, but there were progeny defects at late times in the infection (Fig. 3B and 4A). This late temporal effect of BFA may explain why this commonly used protein transport inhibitor was not previously noted to cause a reduction in chlamydial progeny (7, 8).

We propose that the end of the intracellular infection, marked by the onset of inclusion and host cell lysis, may be the best time point for inhibitor analysis. Less than 10% of total C. trachomatis EB production is completed by 28 hpi (13), and thus measurements of progeny production at or prior to this time point may not accurately represent an overall progeny defect. In addition, progeny assays performed after the onset of host and inclusion lysis may undercount the number of progeny, presumably because EBs from lysed host cells are lost into the supernatant and not recovered. We found that inclusion size and infectious progeny were highest at the time point when host cell lysis was first detected in a few cells by light microscopy. This time point must be experimentally determined because an inhibitor can alter the length of the developmental cycle, as was the case with BFA.

Our findings provide strong justification for the use of a time course analysis as a comprehensive and suitable approach for studying an inhibitor of the Chlamydia infection. The effect of KSK120 in causing an initial delay in inclusion growth, or the late progeny defects with BFA and MK2206 would not have been observed without a time course analysis. In a similar fashion, Clarke and colleagues used a time course analysis to show that the absence of the Chlamydia muridarum plasmid delayed inclusion growth and progeny production (20). Furthermore, Sharma et al. observed that depletion of lipid droplets decreased progeny production at 24 hpi, but increased it at 48 hpi (19). Thus, even though a time course analysis involves more work, it can provide additional information and even mechanistic insights into the effect of an inhibitor on the Chlamydia infection. If a more limited analysis is performed, we propose that the best time point to analyze effects of an inhibitor is at the end of the intracellular infection, at the time of lysis onset, with the understanding that this approach may miss temporal effects.

A key feature of our study is the introduction of Progeny_max_ as a standardized measure to quantify and compare progeny defects in a Chlamydia infection. Progeny_max_ measures maximal production of infectious EBs rather than EBs produced at some interim time point in the intracellular infection. As Progeny_max_ is not measured at a fixed time point, it allows inhibitors to be compared even if they alter the length of the developmental cycle. The concept of Progeny_max_ is generalizable and can be applied to effects on progeny production caused by other experimental interventions, including different growth conditions, chlamydial mutants, and genetic manipulation or protein knockdown of host factors. Our comparison of Progeny_max_ values for several inhibitors revealed that the magnitude of a progeny defect can vary widely, with some inhibitors causing a large, >10-fold defect (Fig. 6). While it is likely that large progeny defects are deleterious *in vivo*, the significance of small progeny defects is less clear. Such smaller defects may require further investigations of the inhibitor in an animal model of Chlamydia infection.

In our studies, we determined Progeny_max_ using the progeny assay, which is a standard assay in the Chlamydia field. While widely used, this assay measures infectious EBs within intact host cells but not EBs that have been released into the supernatant. The supernatant could in principle be analyzed, but the effect of reduced viability after release into inhibitor-containing growth medium at 37°C could be a confounding factor. A related issue is the observation that EBs can be released from an intact host cell by a process called extrusion (4). It is possible that an inhibitor could alter the ratio between inclusion lysis and extrusion, which could be investigated with additional assays (4).

In this study, we used the progeny assay to measure infectious EB production and EM to detect EBs in the inclusion. EB production could also be measured with RT-qPCR or Western blot analyses using EB-specific markers such as *OmcB*, *HctA*, and *HctB*. Immunofluorescence analyses using these same markers could be performed to assess the number of EBs in an infected cell. However, a limitation of these approaches is that they do not provide information about EB infectivity. It is possible that an inhibitor could affect EB maturation but not EB number (26), and such an effect will only be revealed by analysis with a combination of assays.

The use of novel inhibitors revealed that host kinases involved in lipid transport and cholesterol metabolism are involved in the Chlamydia infection. Our studies with MK2206 suggest that Akt-mediated transport of host lipids to the inclusion is necessary for RB-to-EB conversion, but not for chlamydial replication. A previous study reported that disruption of Akt activity caused a defect in inclusion growth at 24 hpi, but did not examine late time points (23). Our experiments showed that inclusions from MK2206-treated cells were small early on but eventually grew to normal size. In contrast, AMPK inhibition appeared to enhance chlamydial replication. Thus, the known role of AMPK in promoting cholesterol catabolism and decreasing lipid synthesis may negatively regulate RB replication by limiting lipid availability. Together, these findings indicate that host lipids and cholesterol may play central roles in both RB replication and RB-to-EB conversion. Further analysis of MK2206 and Compound C may help with the development of novel anti-chlamydial compounds and allow mechanistic investigations into the role of host lipids and cholesterol in the Chlamydia infection.

In summary, we describe an improved approach to investigate the effects of small molecule inhibitors on the intracellular Chlamydia infection. Taking potential effects of inhibitors on the length of the intracellular infection and discordant effects on the inclusion and the chlamydial developmental cycle into account, we propose that inhibitor analysis should be performed with multiple assays at the end of the infection, when host cell lysis has just begun. Additional mechanistic understanding may be obtained with a time course analysis and from comparing effects on different steps in the developmental cycle, such as RB replication and RB-to-EB conversion. Our analysis of two novel inhibitors provides new insights into the roles of host lipids and cholesterol in the Chlamydia infection. Overall, this approach for studying anti-chlamydial inhibitors may lead to new therapeutic strategies for treating the highly prevalent infections caused by this pathogenic bacterium.

## MATERIALS AND METHODS

### Cell culture.

HeLa cell lines were purchased from ATCC. All cells were cultured at 37°C and 5.0% CO2 in Dulbecco’s modified Eagle medium (DMEM) (11995-065; Gibco) supplemented with 10% fetal bovine serum (FBS) (S11550; Atlanta Biologicals).

### Chlamydia infections.

HeLa cell monolayers were infected with C. trachomatis serovar L2, strain L2/434/Bu (ATCC VR902B) in SPG (200 mM sucrose, 20 mM sodium phosphate, and 5 mM glutamate, pH 7.2) at a multiplicity of infection (MOI) of 3 and centrifuged at 700 × *g* for 1 h at room temperature. After centrifugation, the inoculum was replaced with 500 μL of DMEM supplemented with 10% FBS. Infection with RST5 CPAF WT and RST17 CPAF mutant, a generous gift from Dr. Raphael Valdivia (Duke University), were performed following the same infection protocol. Infected cells were harvested for immunofluorescence analysis at stated time points in the developmental cycle.

For inhibitor treatments, small molecule inhibitors were diluted into DMEM supplemented with 10% FBS, followed by adding this inhibitor-containing medium to infected monolayers immediately after the 1-h centrifugation step.

### Infection efficiency.

HeLa cells plated in 24-well dishes were infected with C. trachomatis at an MOI of 3 and treated with an inhibitor starting at 1 hpi. At 36 hpi, samples were fixed in 100% ice-cold methanol, permeabilized, blocked, and stained for MOMP. Using a 63X objective, the number of cells containing an inclusion was quantified from 10 different fields. The data were expressed as the percentage of cells with an inclusion, which indicates infection efficiency.

### Pharmacological compounds.

Pharmacological compounds included 15 μM KSK120 (generous gift from Dr. Sven Bergström, Umeå University, Umeå); 1 μg/mL Brefeldin A, BFA (AAJ62340MA; Fisher Scientific); 3 μM MK2206 (S1078; Selleckchem); 2 μM Compound C (171260; Sigma-Aldrich); 12.5 μM H89 hydrochloride (CAS 130964-39-5; Cayman Chemical). All inhibitors were reconstituted in dimethyl sulfoxide (DMSO).

### Antibodies used in this study.

In this study, primary antibody used was mouse anti-MOMP antibody (generous gift from Ellena Peterson, University of California, Irvine) and secondary antibody was donkey anti-mouse IgG Alexa Fluor 555 (A31570; Invitrogen).

### Immunofluorescence microscopy.

Cells, grown and infected on glass coverslips, were fixed in 100% ice-cold methanol for 10 min at the indicated time points. They were then permeabilized in blocking buffer (2% FBS, 0.1% Triton) for 30 min at room temperature and stained with primary and secondary antibodies to visualize C. trachomatis. Coverslips were mounted with ProLong Glass antifade containing NucBlue to stain DNA (P36985; Invitrogen). Immunofluorescence microscopy images were acquired on a Zeiss Axiovert 200M microscope. Manual tracing using ImageJ software was used to quantify inclusion sizes. One hundred inclusion areas were measured for each condition, from one representative experiment. Data are expressed as means ± standard deviation (SD).

### Progeny assay.

C. trachomatis-infected cells were treated with inhibitor starting at 1 hpi. At the indicated times, cells were washed with 1X PBS and replaced with 500 μL of cold SPG. Infected cells were lysed by freezing at −80°C for 30 min, followed by thawing at 37°C for 15 min. After vortexing vigorously, cell lysates were serially diluted in SPG (without inhibitor) and used for reinfection of HeLa cell monolayers in a 96-well-plate format. At 27 hpi, cells were fixed in 100% ice-cold methanol. Using immunofluorescence microscopy with an antibody to MOMP, the number of inclusion forming units (IFUs) were counted from 5 fields of view using a 20X objective. The number of progeny per cell was calculated by dividing the total number of infectious progeny (IFU/mL) by the number of host cells present at the start of the infection.

Progeny_max_ is defined as the maximal number of infectious progeny detected at the time point that corresponds to the onset of inclusion lysis, defined as the last time point with intact inclusions.

### Quantitative PCR (pPCR).

The number of chlamydial genomes per infected HeLa cell was measured by qPCR. A plasmid encoding the C. trachomatis euo gene was used to generate a standard curve from which we calculated the Chlamydia copy number. PCRs with primers that recognize a host cell gene (GAPDH) were performed to generate a PCR product from which we calculated the total number of host cells. The total number of Chlamydia genomes per cell was determined by normalizing the Chlamydia copy number (EUO) to the respective GAPDH values.

*Primer sequences for EUO*: 5′-TTATTCCGTGGGACAAGTGG-3′ (forward primer) and 5′-TGCAAGACTTTTCCCTTTGC-3′ (reverse primer).

*Primer sequences for GAPDH*: 5′-GGCGCTCACTGTTCTCTCCC-3′ (forward primer) and 5′-CGCAAGGCTCGTAGACGCG-3′ (reverse primer). Each qPCR was conducted using SsoAdvanced universal SYBR green supermix (1725271; Bio-Rad) and was run on a Bio-Rad thermocycler.

### Electron microscopy analysis.

C. trachomatis-infected HeLa cells were washed once with 1X PBS and trypsinized at 37°C for 5 min. Samples were centrifuged at 300 × *g* for 5 min, washed gently with 5 mL of 1X PBS, and centrifuged for another 5 min. Infected cells were resuspended in 1 mL of fixative EM-grade 2% paraformaldehyde (100503-917; VWR), EM-grade 2.5% glutaraldehyde (NC9861069; Fisher Scientific) in 0.1 M cacodylate buffer and incubated at room temperature for 2 h with gentle rocking. After pelleting the cells at 300 × *g* for 5 min, the fixative was removed, replaced with 0.1 M cacodylate buffer, and stored at 4°C. Samples were processed at the Molecular Microbiology Imaging Facility at the Washington University School of Medicine in St. Louis.

### Statistical analysis.

At least three independent biological replicates of each experiment were conducted, and the results are presented as means ± standard error (SE). Data were analyzed by unpaired, two-tailed t tests on GraphPad Prism software, version 9.

10.1128/mbio.01076-22.3 FIG S3 The RST17 CPAF mutant produces modest effects on progeny and inclusion growth. (A) HeLa cells were infected with either the RST17 (CPAF mutant) or its control counterpart RST5 (CPAF WT) strains. A progeny assay time course was used to measure infectious EBs from intact inclusions at the time points indicated. Data are presented as means ± SE (*n *= 3); **, *P ≤ *0.01; ***, *P < *0.001. (B) The number of chlamydial genomes for the CPAF WT and mutant-infected HeLa cells was determined by qPCR at the indicated time points. Data are presented as means ± SE (*n *= 3). **, *P ≤ *0.01; n.s., data not statistically significant. *, *P < *0.05; n.s., data not statistically significant. (C) Immunofluorescence images of CPAF WT or CPAF mutant-infected HeLa cells were fixed at the times indicated and stained with antibodies to MOMP (red) and DAPI (blue). Scale bar is 10 μm. (D) The average inclusion area from 100 inclusions was measured for each condition at each time point. The data from one representative experiment are presented as means ± SD; ***, *P < *0.001; n.s., data not statistically significant. Download FIG S3, TIF file, 0.6 MB. Copyright © 2022 Muñoz et al. 2022 Muñoz et al. https://creativecommons.org/licenses/by/4.0/ This content is distributed under the terms of the Creative Commons Attribution 4.0 International license.
