# Supplementary figures and images for: Differential Effects of Small Molecule Inhibitors on the Intracellular Chlamydia Infection
Source: mBio. 2022 Jun 15;13(4):e01076-22. doi: 10.1128/mbio.01076-22 (PMC9426518; doi:10.1128/mbio.01076-22)

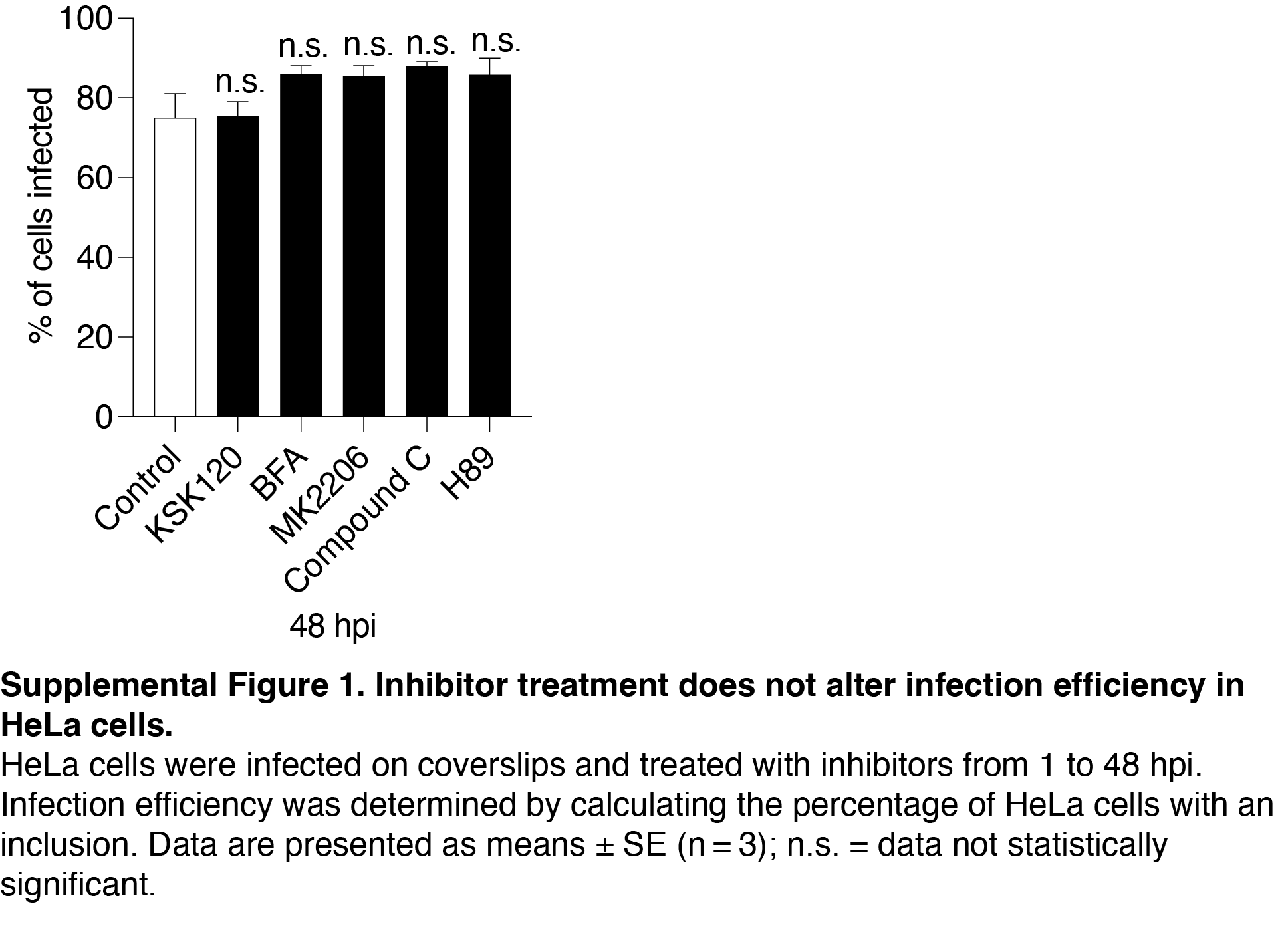

Supplement: FIG S1 [file mbio.01076-22-s0001.tif]
